# BEWARE: Body awareness training in the treatment of wearing-off related anxiety in patients with Parkinson’s disease: study protocol for a randomized controlled trial

**DOI:** 10.1186/s13063-015-0804-0

**Published:** 2015-06-23

**Authors:** Ires Ghielen, Odile A. van den Heuvel, Cees J. T. de Goede, Marieke Houniet-de Gier, Emma H. Collette, Ingrid A. L. Burgers-Bots, Sonja Rutten, Gert Kwakkel, Kees Vermunt, Bep van Vliet, Henk W. Berendse, Erwin E. H. van Wegen

**Affiliations:** Department of Psychiatry, VU University Medical Center, PO Box 7057, 1007 MB Amsterdam, The Netherlands; Department of Anatomy & Neurosciences, VU University Medical Center, Van der Boechorststraat 7, 1081 BT Amsterdam, The Netherlands; Neuroscience Campus Amsterdam, De Boelelaan 1085, 1081 HV Amsterdam, The Netherlands; Department of Rehabilitation Medicine, MOVE Research Institute Amsterdam, VU University Medical Center, De Boelelaan 1118, 1081 HZ Amsterdam, The Netherlands; Department of Medical Psychology, VU University Medical Center, De Boelelaan 1118, 1081 HZ Amsterdam, The Netherlands; Parkinson Vereniging, Postbus 46, 3980 CA Bunnik, The Netherlands; Department of Neurology, VU University Medical Center, De Boelelaan 1117, 1081 HV Amsterdam, The Netherlands

**Keywords:** Parkinson’s disease, Wearing-off, Anxiety, Body awareness, Randomized controlled trial, Self-efficacy

## Abstract

**Background:**

The wearing-off phenomenon in patients with Parkinson’s disease (PD) is a complication of prolonged levodopa usage. During this phenomenon, motor symptoms such as rigidity and freezing re-emerge. This is often accompanied by non-motor symptoms, including anxiety, the so-called wearing-off related anxiety (WRA). Current treatment options are limited and typically focus on either the physical or mental aspects of wearing-off. An integrated approach seems warranted in order to optimally address the complex reciprocal interactions between these aspects. Also, because wearing-off is eventually inescapable, treatment needs to focus on coping, acceptance, and self-efficacy. We therefore developed an integrated body awareness intervention, combining principles from physical therapy with acceptance and commitment therapy to teach patients to deal with WRA. This study will investigate whether this new intervention, named BEWARE, is more effective than treatment as usual in increasing self-efficacy.

**Methods/Design:**

This is a single-blinded randomized controlled trial in 36 PD patients who experience WRA. Subjects will be recruited from the outpatient clinic for movement disorders of the VU University Medical Center. After providing written informed consent, patients will be randomly assigned to an experimental (BEWARE) or treatment-as-usual (physical therapy) group. Clinical assessments will be performed prior to the intervention, directly after the 6-week intervention period, and at 3-month naturalistic follow-up by a blinded investigator not involved in the study. The primary outcome measure is self-efficacy, and secondary outcomes focus on mobility, daily functioning, anxiety, and quality of life.

**Discussion:**

Because wearing-off is an inevitable consequence of levodopa therapy and current treatment options are insufficient, a multidisciplinary intervention that addresses both physical and mental aspects of wearing-off in PD may foster additional benefits for treating WRA in PD patients over mono-disciplinary care alone.

**Trial registration:**

ClinicalTrials.gov identifier: NCT02054845. Date of registration: 30 January 2014.

## Background

Parkinson’s disease (PD) is a progressive neurodegenerative disorder of the central nervous system and has a prevalence of 1.6 % among people who are at least 65 years old [1]. The neurobiological hallmark of PD is a loss of dopaminergic cells, causing the typical motor symptoms such as tremor, rigidity, slowness of movement, postural instability, and freezing. Non-motor symptoms, such as autonomic failure, fatigue, depression, and anxiety are also prevalent and this is likely due to an additional involvement of non-dopaminergic systems [2].

To supplement the shortage of dopamine, levodopa treatment is currently the most applied and effective symptomatic treatment [3]. When the dopamine replacement therapy (DRT) takes effect and symptoms become less prominent, the patient is in the ‘on’ state. In contrast, the state in which the patient is in need of a new dose of dopamine and experiences intensified PD symptoms is referred to as the ‘off’ state. A re-emergence of PD symptoms, shifting from an ‘on’ state to an ‘off’ state, is called wearing-off. This typically occurs prior to the next scheduled dose of dopaminergic therapy taking effect [4] and is related to longer disease duration [5].

Motor and non-motor symptoms have reciprocal influences [6]. About 75 % of patients with motor fluctuations, including wearing-off, experience mood or anxiety fluctuations or both in parallel [7]. This wearing-off related anxiety (WRA) is characterized not only by subjective feelings of anxiety but also by physical complaints, such as sweating, abdominal distress, and shortness of breath. Rutten et al. [8], by performing a factor analysis on the Beck Anxiety Inventory (BAI), showed that anxiety symptoms in PD show significant overlap with both autonomic and motor symptoms. This finding demonstrates that physical and mental symptoms are intertwined in PD.

The physical symptoms accompanying WRA are often incongruent with the actual severity and physical impact of motor symptoms of wearing-off, suggesting an increased sensitivity and reactivity to the occurrence of wearing-off symptoms and heightened body awareness in these patients. Body awareness involves a focus on and awareness of internal bodily sensations [9]. An abnormal increase in body awareness can be maladaptive [9] and is, in general, also related to anxiety disorders [10–14].

The anxiety symptoms experienced by patients with PD are often responsive to dopaminergic medication [15]. Therefore, the first therapeutic approach for treating WRA is to optimize the DRT [16]. As the disease progresses, increasing dopaminergic medication (both frequency and dosage) becomes insufficient and complicated because of the increased occurrence of dyskinesias [17]. Also, random fluctuations appear to be more difficult to treat with pharmacotherapeutic approaches because they are unpredictable and not directly related to a lower level of dopamine [17].

Non-pharmaceutical approaches include exercise programs and physical therapy. These have been shown to improve motor problems, daily functioning, and quality of life in patients with PD [18–21]. Though effective for improving mobility-related problems, current physical rehabilitation approaches typically do not offer tools to address the non-motor symptoms of wearing-off.

Cognitive behavior therapy (CBT), mindfulness, and acceptance and commitment therapies (ACTs) have proven to be effective in reducing anxiety symptoms and avoidance behavior in patients with anxiety disorders, also enhancing quality of life [22, 23]. Therefore, classic tools from CBT and ACT might be useful in the treatment of the debilitating effects of WRA. A reduction of anxiety symptoms in patients with PD is observed in most of the CBT studies [24–26]. However, based on clinical experience, efficacy of classic CBT is limited in PD care because the cognitive methods are suboptimal in addressing the interaction between non-motor and motor symptoms during wearing-off. Moreover, the classic approaches aim to reduce the symptoms, whereas the inevitability of motor and non-motor fluctuations in PD demands the ability to maintain physical and mental balance despite the presence of those fluctuations. This is of great importance mainly in the more advanced stages of PD, in which symptom management is more challenging [3]. Therefore, interventions need to focus on independence and self-efficacy more than on reducing symptoms.

To address both the physical and the mental aspects of PD, Wahbeh et al. [27] reviewed mind-body interventions in the treatment of PD and showed that participating in tai chi classes improved the patients’ physical condition. Landsman-Dijkstra et al. [28] tested a highly structured and standardized 3-day body awareness program in 14 participants who had chronic non-specific psychosomatic symptoms. Increases of body awareness, self-efficacy, and quality of life were found after the intervention. However, the researchers did not implement a control group in this study.

Mindfulness-based therapies have proven to be effective in many patient groups, such as patients with chronic pain, anxiety, and depressive disorders, by improving psychological functions and reducing pain and stress [29, 30], although the mean effect size was small (0.42) [30]. In addition, Åkerblom et al. [31] showed that pain-related acceptance is the strongest mediator in CBT treatment in patients with chronic pain. The authors conclude that targeting of acceptance in treatment may lead to further improvements in outcome measures. It has been shown that CBT is effective in PD patients even after a follow-up period on anxiety and coping measures [32]. Because PD is a chronic disease and is accompanied by both physical and mental symptoms (motor and non-motor symptoms), combining mindfulness-based therapy with physical rehabilitation might be of potential benefit for PD patients with WRA.

BEWARE is an integrated body awareness intervention, combined with ACT, in which mindfulness training is incorporated with physical therapy. We will test the effectiveness of this intervention—compared with treatment as usual (TAU)—in terms of self-efficacy in PD patients with WRA. We hypothesize that the BEWARE intervention will be more effective than physical therapy alone (TAU) in improving self-efficacy.

## Methods/Design

### Study design

**Fig. 1:**
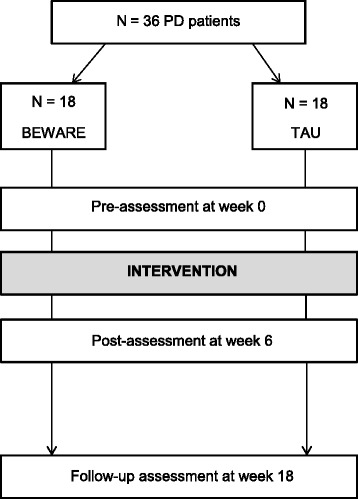
Study flow chart. *BEWARE* body awareness training, *PD* Parkinson’s disease, *TAU* treatment as usual

This will be a single-blind randomized controlled trial. Thirty-six PD patients who experience WRA will be randomly allocated either to the BEWARE training group (*n* = 18, three groups of six patients) or to a control group receiving physical group therapy, which is a usual form of treatment for wearing-off in patients with PD at the VU University Medical Center (*n* = 18, three groups of six patients). Block randomization is done by using concealed opaque envelopes. All participants are asked to maintain the regular medication schedule during the 6-week intervention. Assessments are conducted prior to the intervention and at 6 weeks directly after the intervention. Also, long-term effects will be assessed at 18 weeks of follow-up (from post-enrolment). The assessments will be performed by a blinded investigator who is not involved in the intervention or randomization. Figure 1 demonstrates the study design according to the CONSORT (Consolidated Standards of Reporting Trials) statement [33].

### Patient recruitment

The patients will be recruited from the outpatient clinic of the VU University Medical Center. The neurologists and psychiatrist will be asked to approach PD patients who, in their view, experience WRA. In addition, an announcement will be placed on the website of the Dutch Parkinson patient association (Parkinson Vereniging) and in their magazine. Inclusion and exclusion criteria are listed below.

### Patient inclusion criteria

1. Given a diagnosis of idiopathic PD according to the UK PD Brain Bank criteria [34].
2. Experiencing wearing-off symptoms or response fluctuations (or both), as measured with the Wearing-Off Questionnaire 19 [35]. A patient is considered to be experiencing wearing-off if he or she indicates at least one symptom that improves after taking a next medication dose.
3. Presence of clinically relevant anxiety, defined as a BAI score of more than 26 [36].

### Patient exclusion criteria

1. Other neurological, orthopaedic, or cardiopulmonary problems that in the view of the researchers may interfere with participation.
2. Cognitive impairment defined as a Mini Mental State Examination (MMSE) score of less than 24.
3. Insufficient motivation for participation.

### Intervention

Two treatment conditions are investigated in this study: the experimental condition (BEWARE) and the TAU (physical therapy). Both interventions consist of 12 sessions, each 1 hour long, two times per week for 6 weeks. All treatment sessions occur at the same time of day on the same two days of the week (Monday and Thursday) throughout the study. The treatment groups consist of six patients, in both conditions.

#### Experimental condition: body awareness training (BEWARE)

The experimental intervention is delivered by professionals from the fields of psychology, physical therapy, and psychiatry. The BEWARE training is based mainly on the principles of ACT [37]. The goals of this training are to acquire and apply adequate coping strategies with wearing-off and live a valuable life despite the presence of wearing-off symptoms.

In the first therapy session, psycho-education about PD and wearing-off is discussed by the psychiatrist. In the following sessions, the psychologist explains and provides training in the concepts of body awareness, cognitive defusion, and valued living and prepares the patients for the imaginary exposure. Body awareness is achieved by using attentional exercises. Cognitive defusion techniques attempt to change the way the patient interacts or relates to thoughts, by observing one’s thoughts in a non-judgemental manner. In ACT, valued living is achieved by choosing personal life directions while undermining choices based on avoidance or social compliance.

Imaginary exposure, in ACT therapy known as FEEL (Feeling Experiences Enriches Living) exercises [37], is applied during sessions 6 to 12. During the imaginary exposure, patients are asked to imagine a real-life situation that easily triggers wearing-off. The patients practice with experiencing and daring to allow the feelings that are triggered by the ‘off’ during this imaginary exposure. Throughout these sessions, the ‘off’ situations become more challenging and patients are encouraged to gradually take part in activities that they previously avoided because of the (anticipation of) wearing-off.

The psychological exercises are alternated by physical exercises that are performed by the physical therapist. These exercises include strategies for starting and performing complex movements with risk of falling, such as sitting down and rising from a chair, called transfers. Also, standing, initiation of walking, and walking in complex or stressful situations are addressed. Lastly, moving to rhythmic music helps the patients to relieve stress after the imaginary exposure. These aspects teach the patient to be able to cope with wearing-off situations. To generalize the intended effect, the patients are given homework assignments, such as body awareness exercises and planning value-based committed actions in daily life.

#### Control condition: treatment as usual

The patients in the control group receive TAU based on the current KNGF (Royal Dutch Society for Physical Therapy) guidelines for physical therapy in patients with PD [38]. The group treatment contains exercises for balance, walking, posture, reaching and grasping, strength, flexibility, relaxation, and physical condition. As in the experimental condition, patients are taught strategies on how to make transfers.

### Outcome measures

#### Primary outcome measure

The primary outcome measure is self-efficacy, assessed with the 10-item General Self-Efficacy Scale (GSES) [39]. Self-efficacy is defined as the extent or strength of one’s belief in one’s own ability to complete tasks and reach goals (in other words, a person’s belief in his or her ability to succeed in a particular situation) [40]. In this questionnaire, patients are asked to rate specific statements on a scale from 1 to 4 (1 = not at all true, 2 = hardly true, 3 = moderately true, and 4 = exactly true). The GSES has proven to be a valuable and reliable outcome measure in patients with PD [41].

#### Secondary outcome measures

1. The Parkinson’s Disease Questionnaire-39 (PDQ-39)

This questionnaire is a measure of quality of life adapted for people with PD [42]. It consists of 39 statements that cover eight domains associated with health, such as mobility and emotionality, that can be influenced by PD. Patients can reply by ticking the relevant answer that indicates whether they experienced problems during the past month (five boxes from ‘never’ to ‘always’). The PDQ-39 is a valid and reliable instrument for patients with PD [42, 43].

2. The Wearing-off Questionnaire 19 (WOQ-19)

In this 19-item questionnaire [35], patients with PD indicate which symptoms they experience and whether these symptoms improve after administration of PD medication.

3. Beck Anxiety Inventory (BAI)

This self-report questionnaire consists of 21 common symptoms of anxiety [36]. Patients indicate how much they have been bothered by these symptoms during the past week, including today.

4. Beck Depression Inventory

This 21-item self-report questionnaire measures whether patients experience depressive symptoms [44]. Patients indicate the statements that are most applicable to their own situation during the past week, including today.

5. 10-Meter Walk Test (10MWT)

To measure comfortable walking speed, the patients are asked to walk 10 metres, which are characterized by a starting and a finishing point [45]. The researcher measures the time needed to cover this distance.

6. Timed One-Leg Stance Test (OLST)

This test measures balance by asking the patients to stand on one leg without help [46]. The number of seconds that the patients maintain their balance is noted; the maximum is 60 seconds.

7. The Nottingham Extended Activities of Daily Living (NEADL) index

This 22-item self-report questionnaire covers four aspects of daily living: mobility, activities in the kitchen, domestic tasks, and leisure activities [47]. Patients indicate whether they independently performed activities during the past weeks.

8. Freezing of Gait (FOG) questionnaire

This six-item questionnaire is used to identify current problems with walking and symptoms of freezing [48]. For each item, patients indicate which of the five statements is most applicable.

9. Visual Analogue Scales (VASs)

Before and after each therapy session, patients indicate how they are feeling at the actual moment by using 10 VASs. Patients put a cross on a line of 10 cm with extreme feelings at the edges (for example, ‘relaxed’ or ‘tense’ ,  ‘in control’ or ‘out of control’ , and ‘happy’ or ‘sad’).

### Patient researchers

Because the experimental treatment is a new approach, the opinion and experiences of the participating patients are considered very important. Therefore, two patient researchers of the Dutch Parkinson patient association (Parkinson Vereniging) contribute to the study by anonymously documenting the patients’ expectations and evaluation points for qualitative analysis. Prior to and directly after the intervention period, a semi-structured group interview takes place in which the patients are asked questions related to the study and can share their experiences and suggestions for adaptations in future studies or in the implementation phase. This interview is with the patients (both participants and researchers) only.

### Statistical analysis

#### Calculation of sample size

We expect that, compared with the TAU group, the experimental group will show a 10 % larger improvement on the GSES after treatment. Nieuwboer et al. [49] investigated a 3-week physical home training program and showed that physical measures improved with 4.2–5.5 % compared with 3 weeks of no therapy. Because we expect the effects of the combination of physical therapy and ACT in the BEWARE intervention to be stronger and the intervention period to be longer and more intensive, we hypothesize a larger difference in our primary outcome measure. Considering this study and data on the GSES in other chronic diseases [50], we expect to detect a 10 % larger reduction in GSES score from 32 to 28.8 and an overall standard deviation of 3.3. A minimum of 16 patients is required per arm of the trial. With that, including a dropout of 10 %, we estimate that 36 patients with PD (18 per arm) are needed to achieve a sufficient statistical power of 80 % with two-tailed significance level set at a *P* value of less than 0.05.

#### Data analysis

To promote data quality, double data entry will be used. Multiple imputation will be applied on missing data. Analysis of variance with repeated measures will be used for normally distributed outcomes at interval and ratio level. Friedman’s non-parametric repeated measures test will be applied for outcomes with non-interval or ratio scale level or for non-normally distributed variables. Factors in the analysis will be group (two levels: control and experimental) and time (three levels: baseline, assessment at week 6 (directly post-treatment), and assessment week 18 (3-month follow-up)).

### Ethical considerations

Prior to study participation, written informed consent will be obtained from the patients. This study was approved by the ethics committee of the VU University Medical Center (Medisch Ethische Toetsingscommissie (METc), study number 13.421).

### Data monitoring

The data and safety monitoring board will guard the quality and safety during this study in accordance with the Good Clinical Practice guidelines.

### Discussion (expected results)

Wearing-off is an inevitable and disabling consequence of long-term DRT in patients with PD, and WRA is a concept characterized by a complex reciprocal interaction of motor and non-motor symptoms. The general neglect of these complex interactions in the TAU leaves a gap for treatment innovation. BEWARE is a promising therapy for WRA in PD because it specifically focuses on the interaction between physical and mental symptoms. The specific and unique combination of elements of the therapy aims to increase self-efficacy by strengthening one’s own belief to adequately cope with the wearing off and the disproportional concurrent feelings of anxiety rather than reduce or eliminate symptoms.

Because the development of wearing-off is associated with long-term treatment of PD with DRT as well as with an early onset of disease, longer disease duration, and higher doses of levodopa [5, 17, 51], we will inadvertently include patients who are in a more advanced stage of the disease. Therefore, we must pay specific attention to the potential additional difficulties with patient compliance regarding the amount of effort and commitment we demand from the patients during the twice-weekly intervention.

Considering previous research in combination with our own experience based on a small open pilot study in four patients with PD (results not published), we expect to see a significantly bigger improvement in self-efficacy in the BEWARE condition as compared with the TAU condition. In the pilot study, we observed a decrease in BAI total score in the participating patients. In addition, they reported very positive subjective effects of the intervention. In particular, the group aspect and the holistic approach to address WRA were highly appreciated. Patients stated that they were better able to cope with wearing-off after the intervention period. Considering these first experiences, we believe that BEWARE has the potential to be implemented into health-care practices once its value has been established by using proper testing with high methodological standards.

Because the wearing-off phenomenon is eventually inescapable, this study is of great interest not only from a patient and caregiver perspective but also from a health-care system perspective. Group therapy may lower health-care costs compared with individual treatment because of a reduced therapist/patient ratio, and this treatment may allow patients to function longer and in a more independent way in their own home environment.

## Trial status

Data collection started in January 2014. The expected completion date is August 2015.

## Abbreviations

ACT: Acceptance and commitment therapy
BAI: Beck Anxiety Inventory
BEWARE: Body awareness training in the treatment of wearing-off related anxiety in patients with Parkinson’s disease
CBT: Cognitive behaviour therapy
DRT: Dopamine replacement therapy
GSES: General Self-Efficacy Scale
METc: Medisch Ethische Toetsingscommissie
PD: Parkinson’s disease
PDQ-39: Parkinson’s disease questionnaire-39
TAU: Treatment as usual
VAS: Visual analogue scale
WRA: Wearing-off related anxiety
